# Protestantism a Failure

**Published:** 1869-03

**Authors:** 


### HALL'S

### JOURNAL OF HEALTH.

VOL. XVI. MARCH, 1869. No. 3.

### "PROTESTANTISM A FAILURE,"

WAS lately the subject of some discourses in an Episcopalian pulpit on Fifth Avenue; whereupon a pew-holder announces in an advertisement, that as he has been a Protestant all his life, and has just found out that it is a failure, he has no further use for his pew in that church; and that although it is one of the most "eligible" in the whole building, he will sell it at a very low price.

If Protestantism is a failure from the preacher's stand-point, the Doctor looks at it in a very different light, at least up to the present time, because, in Protestant countries, the average duration of human life is from ten to fifteen years longer than in other parts of the world.

The sunny skies of Italy are famed in "all Greek and Roman story," yet, balmy and beautiful as they are, the people die early. Besides all this, "Protestantism" is but another name for progress, for enterprise, for elevation.

It is very certain that up to the present age, Protestantism has been a grand success in many directions, and if it does not continue to be so, it will not be the fault of Protestant principles, but of the failure of its advocates to live up to them; and if the planet on which we live shall go to moral ruin, Protestantism, as a principle, will stand as the embodiment of a great truth, amid the wreck of matter and the crush of worlds.

"IS PROTESTANTISM SUCCEEDING NOW?"

Is a question of immediate and immense practical importance; is it making as steady and sure a progress as it has done afore-times? are its professors as stern and sterling men and women as they were a hundred or more years ago? There are more members, more preachers, more churches and more of the great things that are always a part and parcel of Protestantism, as the school-house, the college, the hospital, the asylum; but underneath this apparent glory, is there as much sterling doctrinal principle, as much deep-hearted piety, as much implicit reliance on the Divine arm, as much prayerful reading of the Sacred Scriptures, as much holy communion in secret, as much acting from a deep sense of religious principle, as much mourning over inherent and acknowledged sins? To some of these most vital questions, thoughtful men will feel compelled, sorrowfully indeed, to give a negative answer; and to this extent,

"PROTESTANTISM IS GOING BACKWARD."

The price of true religion is eternal vigilance, as well as it is the price of liberty; and as Protestantism and a longer average of human life appear to be in some way connected, the general subject is not altogether out of place in a magazine devoted to the health of the people; but in speaking of it further, some subjects will be introduced which may be thought either "far-fetched," or inappropriate to a publication which goes to the center-tables of refined families and the libraries of our public schools. But events carry us onward, sometimes, whether we will or not; and it may be increasing years have blunted our sensibility. The human machine is made to go of itself, and if not interfered with will be a perpetual motion, at least for a hundred years; but if you have to be introducing tonics into the stomach every once in a while, to help it do its work, or if the liver has to be "waked up" from its torpor, now and then, or expedients ever changing, ever new, have to be devised and adopted to keep the machine in motion, it is proof-positive that "something is rotten in Denmark," that "there is something wrong in the upper story" or elsewhere.

If we have to be giving a man medicine all the time to keep him alive, or rather if he will live in spite of the horrid stuff, it certainly does not prove that there is no man at all, but it does prove that there is something which needs looking into.

### "NEW MEASURES."

This was the battle cry which rent asunder the great Presbyterian Church some thirty or more years ago, and that rent has never been healed; it meant that means were adopted to induce persons to make a profession of religion which were not known to the fathers, which were not sanctioned by Scripture, that they were of doubtful utility, dangerous in their tendencies and pernicious in their effects. That is, they thought the Lord could carry on his own work without the aid of new-fangled notions. This is the way that "Bob Breckenridge" and all the other John Knoxes talked, while a host of noble persons, men of might they were too, such as the Nelsons, and Gallahers, and Hatfields, and Burchards, and Brainards, and Spauldings, and Bullards, and others like them, ranged themselves under the "New Measure" banners, went bravely to work, gathered in their thousands and hundreds of thousands, dying gloriously with their harness on, most of them, believing in the "protracted meetings," the "anxious seat" and others of the "new measures" as legitimate instrumentalities for the conversion of souls. But, alas, it is with doctors of divinity as with doctors of physic, a new measure or a new medicine does very well for a while, but it too often happens that after a season it won't work, it ceases to have its legitimate effect, and some new expedient has to be devised and resorted to, to accomplish the desired result. We, as a doctor, think that medicine becomes inefficient because it is not natural, or, if you please, not the legitimate means of alleviation, resuscitation, and replenishment of the vital forces. Air, exercise, sleep, food, these are natural agencies, and if judiciously used are always safe, are always promotive of good results, and never can be otherwise; and this is the case in all ages, among all peoples, and will be to the end of time. Then we can be only safe in the use of means to replenish, to resuscitate, to fill up the church, to fill up heaven with saved, immortal souls, by using such means of "conversion" as are natural, hence, will be safe and efficient, as long as the world stands, being expressly "ordained of God," and if Protestants adopt other measures, they may seem to do good for awhile, but will be eventually abandoned, while the work which seemed to be accomplished thereby will be as wood, as stubble, which the fires of the judgment will consume to ashes.

Before we state the means for the conversion of the world, which are expressly ordained of God, some statements will be made which may grieve many true Christian hearts, but the surgeon must strike to the core, the knife must go down to the bone, if a certain, speedy and radical cure is to be effected; many a time the filthy, inefficient and hurtful poultice must be instantly torn away, in order to apply the true healing balm.

If a new measure is adopted and is eventually allowed to fall into disuse, it would seem that it was not a natural, or legitimate measure.

If a measure is adopted to prevent a certain state of things among others, yet fails to do so, or that state is aggravated, instead of abated, it would seem that the measure so adopted was not legitimate, was not natural.

Some of the lines which follow are written with great hesitancy; hence, we do not wish to be considered as making a positive statement, but as merely making a suggestive inquiry, as a means of setting the people to think.

In the days of our sunny childhood, when breathing was a bliss, so beautifully and healthfully did the bodily machine work in the out-door street and field and pasture, when we played marbles, gathered walnuts, picked berries and went in swimming, we used to attend, as a matter of course, a prayer-meeting, held on the first Monday afternoon or evening of every month; it was called "The Monthly Concert," adopted as a means of interesting the people on the subject of sending the Gospel to the heathen. It was well attended, at first, slimly afterwards, and finally so few came that it was thought that men could not spare the time out of their own six days, and that if a slice could be taken out of the Lord's day for that purpose, he might not notice it, or, at least, would not interpose any serious objections to the step, while man would be the gainer and kill two birds with one stone; that is, he would worship the Lord and pray for the heathen at the same time. But "all of a sudden" it occurred to us, in thinking out this article, to inquire of a sterling Presbyterian lady at our side, what has become of the monthly concert of prayer? She said she really did not know. Is it in any of the splendid churches on Fifth Avenue? or has it died away to the solitary cabin of some old mother in Israel, who on the banks of some western river, mournfully chants her "Ichabod," as she observes it alone, while the lonely echo answers "Lost!"

Are the protracted meetings and anxious seats also things of the past, and the "mourners benches," where are they? like the deserted ship amid the icebergs of far northern seas, without a solitary occupant. "The Daily Prayer Meeting" was introduced some twelve years ago, as a means of drawing the attention of men to the subject of religion and swept over the continent like wildfire, and already it is a thing of the past in many places at which it was observed with great religious zeal. Within that time a plan was in part carried out, by which every house in every block was to be visited by prudent persons, with a view to getting those to attend church who had not any place of stated worship.

Then there are prayer-meetings once a year for colleges, and later, "a week of prayer" once a year. There is also an organization including some of our wealthiest, most intelligent and most influential men, who are endeavoring to induce Christians of every name to enlist under one banner and make a common cause against the powers of evil, so as to show that the Christian religion with its numerons sects, is many like the billows, but like the ocean, one! It seems that many measures have had their day and have passed into comparative oblivion, and many more perhaps will have the same history. But the failure of these or any others to sustain and advance religion, another name for true Protestantism, does not prove Protestantism a failure, but only that Protestants, from worldly-mindedness or other cause, have not consistently carried out legitimate measures, as some of the above undoubtedly are. But taking all the measures together, have they or have they not failed to intensify and spread a true, active, abiding, Christian principle? With all their appliances, has the number encouragingly increased who have one faith and one practice? But while there is one man in the church who acts thus, there are too many who do otherwise. They too, inquire of the prophets, as many in the every-day affairs of life ask advice and then follow it only in proportion as it meets their previous views. Many want to know what the Bible says, but when they have found that it plainly forbids the course they desire to pursue, they begin to evade it by endeavoring to make it mean something else, or that in their peculiar case it does not apply. When David wanted the pretty wife of Uriah, he set about devising a plan to have the unfortunate Hittite killed, according to red-tape, so that he might not be a murderer in the eyes of the law.

And now comes a truth pertaining to human health and human life which will make every Protestant cheek tingle with shame, because it shows that the means used by Protestants to bring the public mind under the influence of a true Christian principle are now failing; that there is something or other connected with the means used to propagate true religious truth, which make men range themselves under the name of Protestants, while they practically deny the binding influence of its principles, its doctrines; in other words, there is in all the sects a want of indoctrination, as to the fundamental principles of a true religion for example

"MULTIPLY AND REPLENISH THE EARTH,"

Is the express command of Scripture, and yet letters are written every day of the world to eminent physicians, by newly married persons, stating that raising children is too expensive and troublesome, and they want to know how to prevent it: others inquire how to get rid of the unborn child; and they make "putting it away" a matter of dollars and cents, a mere mercantile transaction; and one of the most princely mansions, in the finest street on the globe, was built by the price of this "putting away;" we have seen the house and its occupant and owner. This money comes mainly from Protestant pockets. It may be that Protestants do these things because they do not consider it wrong; this is because they never have had any teachings on the subject; it may be a sin of ignorance, but then it shows that Protestants have failed by their teachings to prevent murder most foul. The Roman Catholics inculcate the doctrine most explicitly, that man becomes an immortal soul at the first instant of conception, and that its destruction at any moment thereafter is murder to all intents and purposes. The disproportion between the Roman Catholic births and Protestant births, in some parts of New England, has become so striking, that fears are entertained of the extinction of the native element; and we have seen the draft of a society for the encouragement of marriage. That is, one part are trying to hire others to get all the babies, instead of having them themselves, doing their duty by proxy, as we hire ministers and choristers that we may pray and sing by proxy.

In our own New York City, with all its splendor and wealth and benevolence, the killing of new-born children, or which is in effect the same thing, leaving them to die in baskets at our doors has become so common, so notorious, so appalling in its frequency, that the *New York Express* of February 2d, 1869, announces that a lady of wealth and benevolence offers ten thousand dollars, as the nucleus of a fund to provide for the taking in and caring for new-born children, from whomsoever may present them, without asking any questions.

The *New York Observer* states, as a matter of personal observation, that human life is safer in the streets of

PARIS AND MADRID, than in New York City, and it would seem too true, in view of the fact that a man is murdered at his own door, in day-light, and the murderer is not apprehended; another man sits quietly in his own room, looking out on the street, when three men enter, rob him of ten thousand dollars, and no trace of them is to be had, and this, before the day-light is gone and while the streets are full of people; on the same day, a man dashes through a bank window-glass, grabs up ten thousand dollars, and all in the face of noon-day. Can things be worse than this in Roman Catholic countries? Is it not known that a controlling number of men in the Legislatures of New York and Pennsylvania and in the National Capitol itself, have their price, and that price is known. Is it not an admitted fact that the Legislature of New Jersey has been carried in the breeches pocket of the Camden and Amboy Railroad Company for many years. Can there be, on the habitable globe, a deeper depth of infamy than is found in the stock-jobbing money operations of New York and in the grain market of Chicago? How much more horrible was "The Inquisition" than has been the conduct of our government, in the persons of its agents, towards the Indians of this country?

But let us stop these sad details and inquire what will make

PROTESTANTISM A SUCCESS, and thus make the average age of man, not twenty as it once was, nor forty as it is now, but as it ought to be, at least one hundred years. Let not the answer be theoretical or a matter of human wisdom; to the law and to the testimony, that is the tribunal to which we appeal for final judgment, and by its decisions we will stand or fall. We need not give the chapter and the verse, but will quote from memory, and what saith the Scripture?

"Preach the Gospel to every creature; go ye out into the highways and hedges and compel them to come in. It shall accomplish that which I please," is said by divinity, shall be the effect of the preached word, until the knowledge of the Lord shall cover the earth as the waters cover the face of the great deep.

All this implies three things:

You must have preachers.

You must have churches.

You must have those churches full of people to hear the preaching.

And with a godly minister, and numerous churches filled with listening multitudes, Christians themselves, at the same time, being instant in season and out of season; praying with all prayer and supplication for the application of the preached word to every hearer by the power of the Spirit, can it be possible that the word of the Lord shall not have free course and be glorified, until all men shall come to the knowledge of the truth?

Here then, we have the whole thing in a nut-shell; good men preaching to crowded houses. To have good men, Boanerges, true sons of thunder, the church must insure salaries which will command the highest talent; for as to this world, God's government is a moral government, conducted by human instrumentalities. There must be no blind leaders of the blind; mind must come in contact with mind; and the logic of fact and reason and feeling, all must be brought to bear, to the convincing of the unconvinced; to make the blind see; the deaf hear, and the hard-hearted to feel; to do this it is necessary to have learning, intellect, culture; it was not intended to advance Christianity by a miracle. The Almighty governs the universe of mind like that of matter, by fixed laws, and through their legitimate operation does he intend to throw around this world a chain of love, and draw it up to heaven, to immortality, and eternal life; nor does he seemingly intend to do this himself; it appears to be his plan to make his church, his children, work their own passage across the sea of time, and to help one another to attain a beatific state beyond the boundaries of this present existence, for it is expressly commanded to "work while it is day;" yet, at the same time, we are to be regarded as, in a sense, "co-workers" with him in building up the heavenly kingdom.

But of the three ordained instrumentalities, preachers, churches, hearers, the two former are attainable with comparative ease; the most difficult is to obtain hearers; a parable was spoken to indicate mainly this important truth, the difficulty of getting hearers and the duty of the church in this regard, which is, to "compel them to come in;" the churches ought to be filled with listeners every Sunday, every time the doors are opened, even if it be necessary to crowd them with the out-casts of earth; and now comes the pith of our story,

THE FAULT AND THE REMEDY.

Hew off all the pew doors, and then let every Christian man and woman not only go to church themselves, but see to it that servants and children and neighbors go along, so that there may not be one single empty seat at the Gospel feast, because the Lord of it is determined that the seats shall be full, that the wedding shall be furnished with guests.

Have an able ministry, liberally supported.

Have free churches filled with hearers.

Have a co-operative praying membership.

These are "measures" as old as the hills, are, always have been, and always will be efficient, having been ordained of God, and will be

"Forever charming
And forever new."

Other things it may be well to have done, but these certainly ought not to be left undone; then will the Western doggerel be written on every banner,

"Ho, every mother's son and daughter,
Here's the Gospel, free as water."

